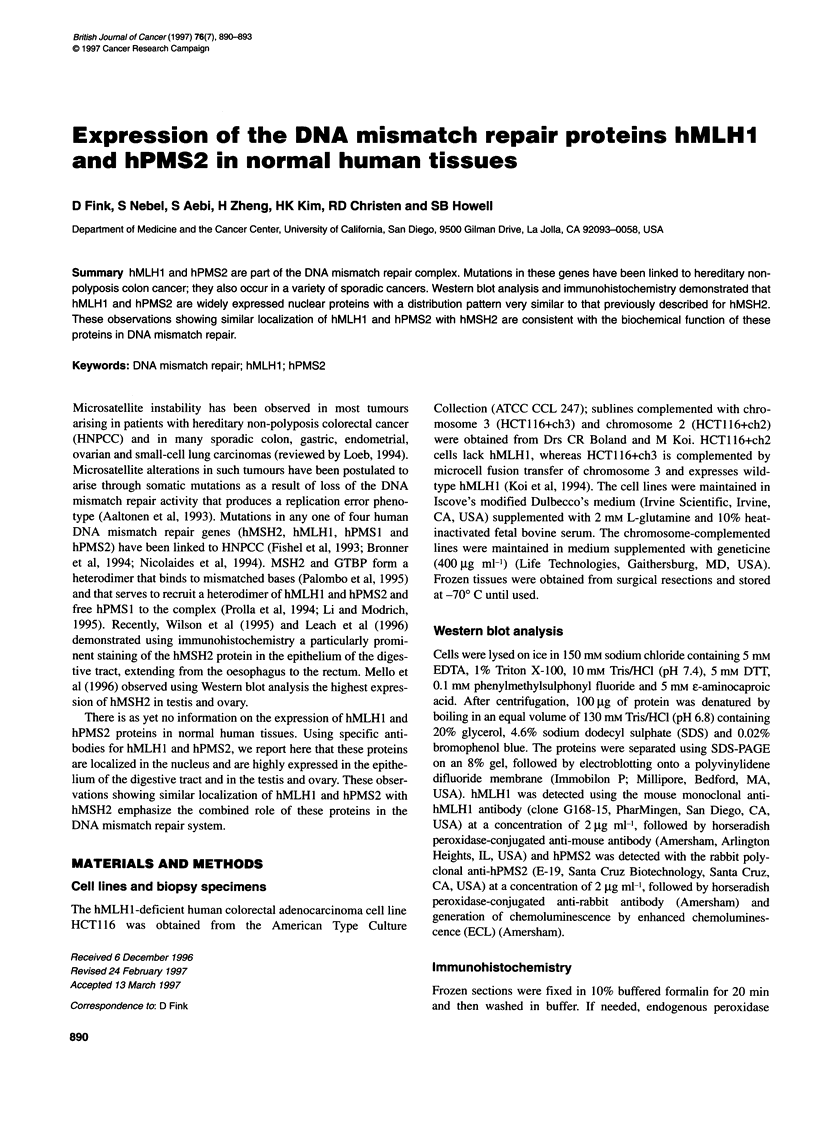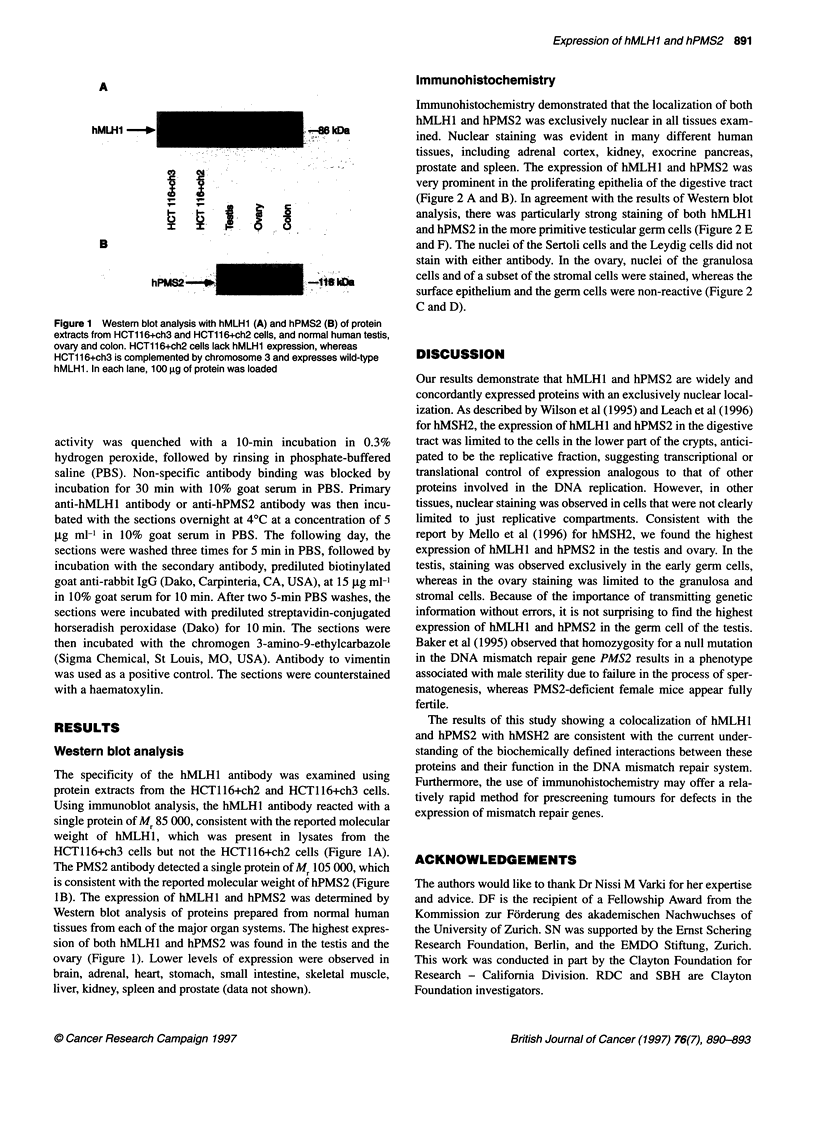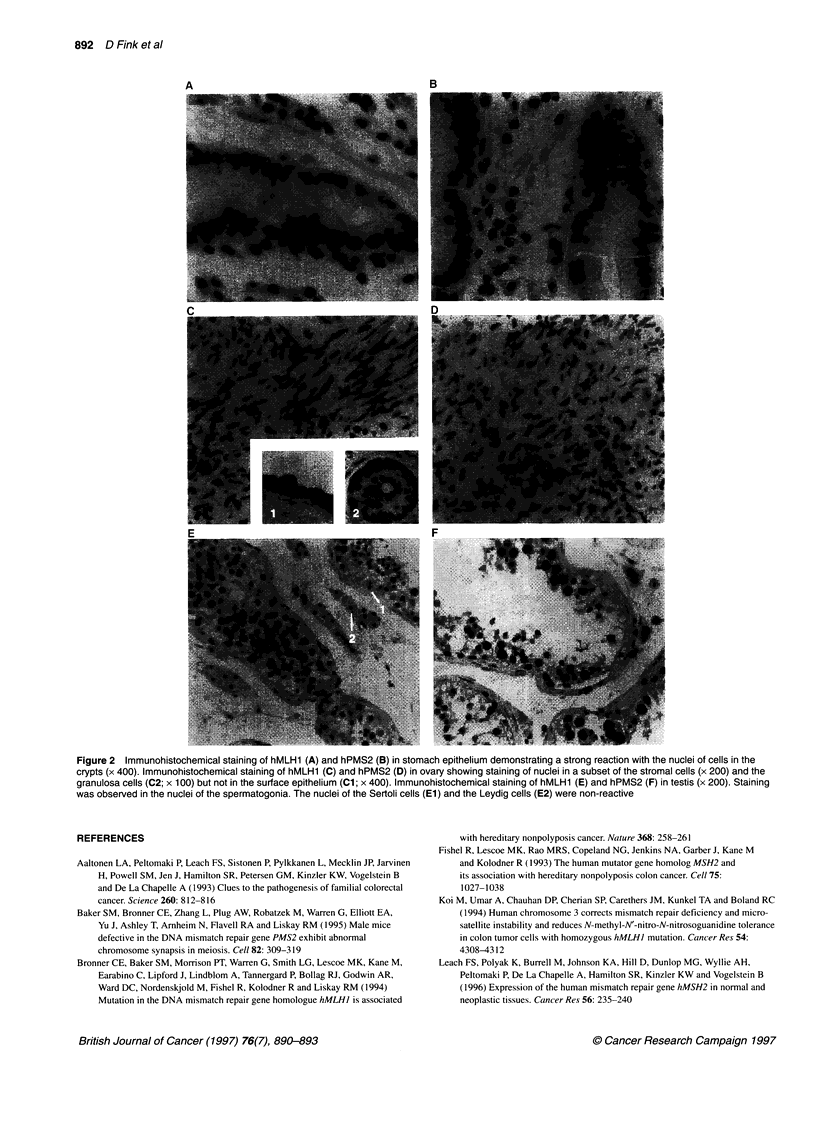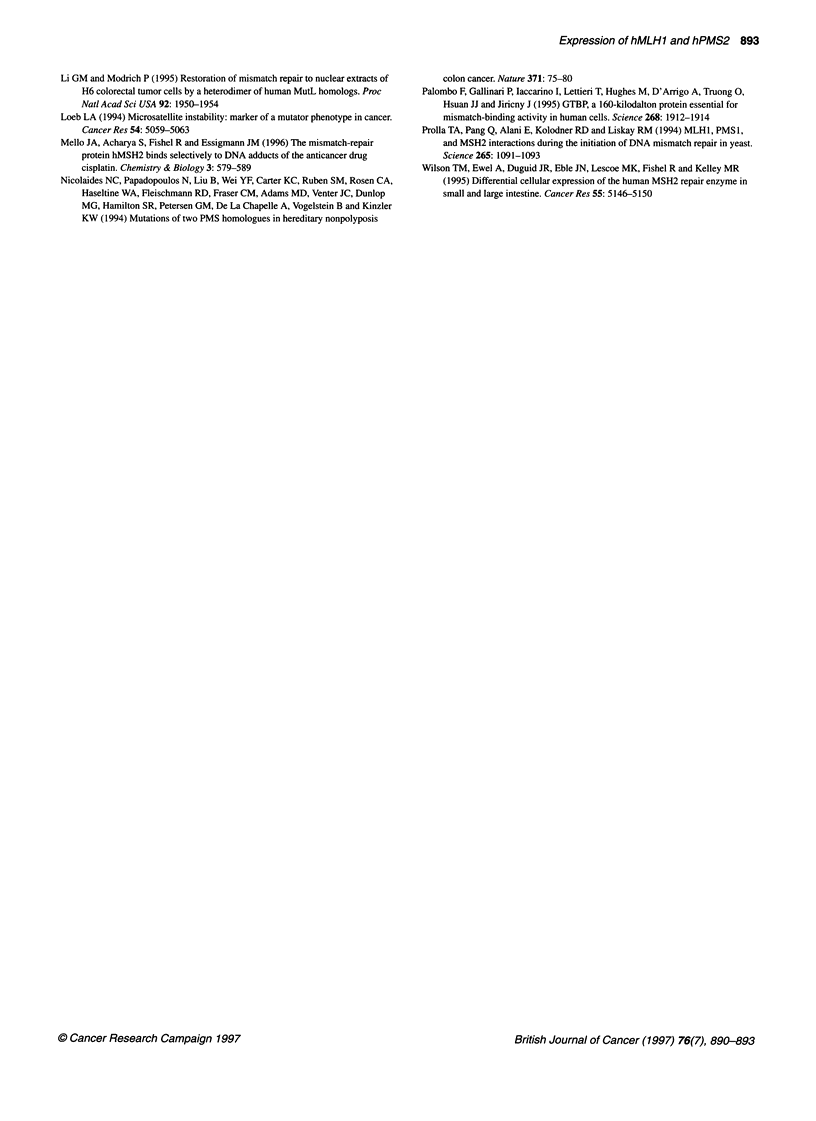# Expression of the DNA mismatch repair proteins hMLH1 and hPMS2 in normal human tissues.

**DOI:** 10.1038/bjc.1997.480

**Published:** 1997

**Authors:** D. Fink, S. Nebel, S. Aebi, H. Zheng, H. K. Kim, R. D. Christen, S. B. Howell

**Affiliations:** Department of Medicine and the Cancer Center, University of California, San Diego, La Jolla 92093-0058, USA.

## Abstract

**Images:**


British Journal of Cancer (1997) 76(7), 890–893
© 1997 Cancer Research Campaign

### Expression of the DNA mismatch repair proteins hMLH1 and hPMS2 in normal human tissues

**D Fink, S Nebel, S Aebi, H Zheng, HK Kim, RD Christen and SB Howell**

Department of Medicine and the Cancer Center, University of California, San Diego, 9500 Gilman Drive, La Jolla, CA 92093–0058, USA

**Summary** hMLH1 and hPMS2 are part of the DNA mismatch repair complex. Mutations in these genes have been linked to hereditary non-polyposis colon cancer; they also occur in a variety of sporadic cancers. Western blot analysis and immunohistochemistry demonstrated that hMLH1 and hPMS2 are widely expressed nuclear proteins with a distribution pattern very similar to that previously described for hMSH2. These observations showing similar localization of hMLH1 and hPMS2 with hMSH2 are consistent with the biochemical function of these proteins in DNA mismatch repair.

**Keywords:** DNA mismatch repair; hMLH1; hPMS2

Microsatellite instability has been observed in most tumours arising in patients with hereditary non-polyposis colorectal cancer (HNPCC) and in many sporadic colon, gastric, endometrial, ovarian and small-cell lung carcinomas (reviewed by Loeb, 1994). Microsatellite alterations in such tumours have been postulated to arise through somatic mutations as a result of loss of the DNA mismatch repair activity that produces a replication error phenotype (Aaltonen et al, 1993). Mutations in any one of four human DNA mismatch repair genes (hMSH2, hMLH1, hPMS1 and hPMS2) have been linked to HNPCC (Fishel et al, 1993; Bronner et al, 1994; Nicolaides et al, 1994). MSH2 and GTBP form a heterodimer that binds to mismatched bases (Palombo et al, 1995) and that serves to recruit a heterodimer of hMLH1 and hPMS2 and free hPMS1 to the complex (Prolla et al, 1994; Li and Modrich, 1995). Recently, Wilson et al (1995) and Leach et al (1996) demonstrated using immunohistochemistry a particularly prominent staining of the hMSH2 protein in the epithelium of the digestive tract, extending from the oesophagus to the rectum. Mello et al (1996) observed using Western blot analysis the highest expression of hMSH2 in testis and ovary.

There is as yet no information on the expression of hMLH1 and hPMS2 proteins in normal human tissues. Using specific antibodies for hMLH1 and hPMS2, we report here that these proteins are localized in the nucleus and are highly expressed in the epithelium of the digestive tract and in the testis and ovary. These observations showing similar localization of hMLH1 and hPMS2 with hMSH2 emphasize the combined role of these proteins in the DNA mismatch repair system.

### MATERIALS AND METHODS

### Cell lines and biopsy specimens

The hMLH1-deficient human colorectal adenocarcinoma cell line HCT116 was obtained from the American Type Culture Collection (ATCC CCL 247); sublines complemented with chromosome 3 (HCT116+ch3) and chromosome 2 (HCT116+ch2) were obtained from Drs CR Boland and M Koi. HCT116+ch2 cells lack hMLH1, whereas HCT116+ch3 is complemented by microcell fusion transfer of chromosome 3 and expresses wild-type hMLH1 (Koi et al, 1994). The cell lines were maintained in Iscove's modified Dulbecco's medium (Irvine Scientific, Irvine, CA, USA) supplemented with 2 mM L-glutamine and 10% heat-inactivated fetal bovine serum. The chromosome-complemented lines were maintained in medium supplemented with geneticine (400 μg $ml^{-1}$) (Life Technologies, Gaithersburg, MD, USA). Frozen tissues were obtained from surgical resections and stored at –70° C until used.

### Western blot analysis

Cells were lysed on ice in 150 mM sodium chloride containing 5 mM EDTA, 1% Triton X-100, 10 mM Tris/HCl (pH 7.4), 5 mM DTT, 0.1 mM phenylmethylsulphonyl fluoride and 5 mM ε-aminocaproic acid. After centrifugation, 100 μg of protein was denatured by boiling in an equal volume of 130 mM Tris/HCl (pH 6.8) containing 20% glycerol, 4.6% sodium dodecyl sulphate (SDS) and 0.02% bromophenol blue. The proteins were separated using SDS-PAGE on an 8% gel, followed by electroblotting onto a polyvinylidene difluoride membrane (Immobilon P; Millipore, Bedford, MA, USA). hMLH1 was detected using the mouse monoclonal anti-hMLH1 antibody (clone G168-15, PharMingen, San Diego, CA, USA) at a concentration of 2 μg $ml^{-1}$, followed by horseradish peroxidase-conjugated anti-mouse antibody (Amersham, Arlington Heights, IL, USA) and hPMS2 was detected with the rabbit polyclonal anti-hPMS2 (E-19, Santa Cruz Biotechnology, Santa Cruz, CA, USA) at a concentration of 2 μg $ml^{-1}$, followed by horseradish peroxidase-conjugated anti-rabbit antibody (Amersham) and generation of chemoluminescence by enhanced chemoluminescence (ECL) (Amersham).

### Immunohistochemistry

Frozen sections were fixed in 10% buffered formalin for 20 min and then washed in buffer. If needed, endogenous peroxidase

*Received 6 December 1996*
*Revised 24 February 1997*
*Accepted 13 March 1997*

*Correspondence to:* D Fink

**Figure 1** Western blot analysis with hMLH1 (**A**) and hPMS2 (**B**) of protein extracts from HCT116+ch3 and HCT116+ch2 cells, and normal human testis, ovary and colon. HCT116+ch2 cells lack hMLH1 expression, whereas HCT116+ch3 is complemented by chromosome 3 and expresses wild-type hMLH1. In each lane, 100 μg of protein was loaded activity was quenched with a 10-min incubation in 0.3% hydrogen peroxide, followed by rinsing in phosphate-buffered saline (PBS). Non-specific antibody binding was blocked by incubation for 30 min with 10% goat serum in PBS. Primary anti-hMLH1 antibody or anti-hPMS2 antibody was then incubated with the sections overnight at 4°C at a concentration of 5 μg $ml^{-1}$ in 10% goat serum in PBS. The following day, the sections were washed three times for 5 min in PBS, followed by incubation with the secondary antibody, prediluted biotinylated goat anti-rabbit IgG (Dako, Carpinteria, CA, USA), at 15 μg $ml^{-1}$ in 10% goat serum for 10 min. After two 5-min PBS washes, the sections were incubated with prediluted streptavidin-conjugated horseradish peroxidase (Dako) for 10 min. The sections were then incubated with the chromogen 3-amino-9-ethylcarbazole (Sigma Chemical, St Louis, MO, USA). Antibody to vimentin was used as a positive control. The sections were counterstained with a haematoxylin.

### RESULTS

### Western blot analysis

The specificity of the hMLH1 antibody was examined using protein extracts from the HCT116+ch2 and HCT116+ch3 cells. Using immunoblot analysis, the hMLH1 antibody reacted with a single protein of $M_r$ 85 000, consistent with the reported molecular weight of hMLH1, which was present in lysates from the HCT116+ch3 cells but not the HCT116+ch2 cells (Figure 1A). The PMS2 antibody detected a single protein of $M_r$ 105 000, which is consistent with the reported molecular weight of hPMS2 (Figure 1B). The expression of hMLH1 and hPMS2 was determined by Western blot analysis of proteins prepared from normal human tissues from each of the major organ systems. The highest expression of both hMLH1 and hPMS2 was found in the testis and the ovary (Figure 1). Lower levels of expression were observed in brain, adrenal, heart, stomach, small intestine, skeletal muscle, liver, kidney, spleen and prostate (data not shown).

### Immunohistochemistry

Immunohistochemistry demonstrated that the localization of both hMLH1 and hPMS2 was exclusively nuclear in all tissues examined. Nuclear staining was evident in many different human tissues, including adrenal cortex, kidney, exocrine pancreas, prostate and spleen. The expression of hMLH1 and hPMS2 was very prominent in the proliferating epithelia of the digestive tract (Figure 2 A and B). In agreement with the results of Western blot analysis, there was particularly strong staining of both hMLH1 and hPMS2 in the more primitive testicular germ cells (Figure 2 E and F). The nuclei of the Sertoli cells and the Leydig cells did not stain with either antibody. In the ovary, nuclei of the granulosa cells and of a subset of the stromal cells were stained, whereas the surface epithelium and the germ cells were non-reactive (Figure 2 C and D).

### DISCUSSION

Our results demonstrate that hMLH1 and hPMS2 are widely and concordantly expressed proteins with an exclusively nuclear localization. As described by Wilson et al (1995) and Leach et al (1996) for hMSH2, the expression of hMLH1 and hPMS2 in the digestive tract was limited to the cells in the lower part of the crypts, anticipated to be the replicative fraction, suggesting transcriptional or translational control of expression analogous to that of other proteins involved in the DNA replication. However, in other tissues, nuclear staining was observed in cells that were not clearly limited to just replicative compartments. Consistent with the report by Mello et al (1996) for hMSH2, we found the highest expression of hMLH1 and hPMS2 in the testis and ovary. In the testis, staining was observed exclusively in the early germ cells, whereas in the ovary staining was limited to the granulosa and stromal cells. Because of the importance of transmitting genetic information without errors, it is not surprising to find the highest expression of hMLH1 and hPMS2 in the germ cell of the testis. Baker et al (1995) observed that homozygosity for a null mutation in the DNA mismatch repair gene *PMS2* results in a phenotype associated with male sterility due to failure in the process of spermatogenesis, whereas PMS2-deficient female mice appear fully fertile.

The results of this study showing a colocalization of hMLH1 and hPMS2 with hMSH2 are consistent with the current understanding of the biochemically defined interactions between these proteins and their function in the DNA mismatch repair system. Furthermore, the use of immunohistochemistry may offer a relatively rapid method for prescreening tumours for defects in the expression of mismatch repair genes.

### ACKNOWLEDGEMENTS

The authors would like to thank Dr Nissi M Varki for her expertise and advice. DF is the recipient of a Fellowship Award from the Kommission zur Förderung des akademischen Nachwuchses of the University of Zurich. SN was supported by the Ernst Schering Research Foundation, Berlin, and the EMDO Stiftung, Zurich. This work was conducted in part by the Clayton Foundation for Research – California Division. RDC and SBH are Clayton Foundation investigators.

*© Cancer Research Campaign 1997*

**Figure 2** Immunohistochemical staining of hMLH1 (**A**) and hPMS2 (**B**) in stomach epithelium demonstrating a strong reaction with the nuclei of cells in the crypts (× 400). Immunohistochemical staining of hMLH1 (**C**) and hPMS2 (**D**) in ovary showing staining of nuclei in a subset of the stromal cells (× 200) and the granulosa cells (**C2**; × 100) but not in the surface epithelium (**C1**; × 400). Immunohistochemical staining of hMLH1 (**E**) and hPMS2 (**F**) in testis (× 200). Staining was observed in the nuclei of the spermatogonia. The nuclei of the Sertoli cells (**E1**) and the Leydig cells (**E2**) were non-reactive

### REFERENCES

Aaltonen LA, Peltomaki P, Leach FS, Sistonen P, Pylkkanen L, Mecklin JP, Jarvinen H, Powell SM, Jen J, Hamilton SR, Petersen GM, Kinzler KW, Vogelstein B and De La Chapelle A (1993) Clues to the pathogenesis of familial colorectal cancer. *Science* **260**: 812–816

Baker SM, Bronner CE, Zhang L, Plug AW, Robatzek M, Warren G, Elliott EA, Yu J, Ashley T, Arnheim N, Flavell RA and Liskay RM (1995) Male mice defective in the DNA mismatch repair gene *PMS2* exhibit abnormal chromosome synapsis in meiosis. *Cell* **82**: 309–319

Bronner CE, Baker SM, Morrison PT, Warren G, Smith LG, Lescoe MK, Kane M, Earabino C, Lipford J, Lindblom A, Tannergard P, Bollag RJ, Godwin AR, Ward DC, Nordenskjold M, Fishel R, Kolodner R and Liskay RM (1994) Mutation in the DNA mismatch repair gene homologue *hMLH1* is associated with hereditary nonpolyposis cancer. *Nature* **368**: 258–261

Fishel R, Lescoe MK, Rao MRS, Copeland NG, Jenkins NA, Garber J, Kane M and Kolodner R (1993) The human mutator gene homolog *MSH2* and its association with hereditary nonpolyposis colon cancer. *Cell* **75**: 1027–1038

Koi M, Umar A, Chauhan DP, Cherian SP, Carethers JM, Kunkel TA and Boland RC (1994) Human chromosome 3 corrects mismatch repair deficiency and microsatellite instability and reduces *N*-methyl-*N*′-nitro-*N*-nitrosoguanidine tolerance in colon tumor cells with homozygous *hMLH1* mutation. *Cancer Res* **54**: 4308–4312

Leach FS, Polyak K, Burrell M, Johnson KA, Hill D, Dunlop MG, Wyllie AH, Peltomaki P, De La Chapelle A, Hamilton SR, Kinzler KW and Vogelstein B (1996) Expression of the human mismatch repair gene *hMSH2* in normal and neoplastic tissues. *Cancer Res* **56**: 235–240

© Cancer Research Campaign 1997

Li GM and Modrich P (1995) Restoration of mismatch repair to nuclear extracts of H6 colorectal tumor cells by a heterodimer of human MutL homologs. *Proc Natl Acad Sci USA* **92**: 1950–1954

Loeb LA (1994) Microsatellite instability: marker of a mutator phenotype in cancer. *Cancer Res* **54**: 5059–5063

Mello JA, Acharya S, Fishel R and Essigmann JM (1996) The mismatch-repair protein hMSH2 binds selectively to DNA adducts of the anticancer drug cisplatin. *Chemistry & Biology* **3**: 579–589

Nicolaides NC, Papadopoulos N, Liu B, Wei YF, Carter KC, Ruben SM, Rosen CA, Haseltine WA, Fleischmann RD, Fraser CM, Adams MD, Venter JC, Dunlop MG, Hamilton SR, Petersen GM, De La Chapelle A, Vogelstein B and Kinzler KW (1994) Mutations of two PMS homologues in hereditary nonpolyposis colon cancer. *Nature* **371**: 75–80

Palombo F, Gallinari P, Iaccarino I, Lettieri T, Hughes M, D'Arrigo A, Truong O, Hsuan JJ and Jiricny J (1995) GTBP, a 160-kilodalton protein essential for mismatch-binding activity in human cells. *Science* **268**: 1912–1914

Prolla TA, Pang Q, Alani E, Kolodner RD and Liskay RM (1994) MLH1, PMS1, and MSH2 interactions during the initiation of DNA mismatch repair in yeast. *Science* **265**: 1091–1093

Wilson TM, Ewel A, Duguid JR, Eble JN, Lescoe MK, Fishel R and Kelley MR (1995) Differential cellular expression of the human MSH2 repair enzyme in small and large intestine. *Cancer Res* **55**: 5146–5150

© Cancer Research Campaign 1997